# Anomalous Near-Surface Low-Salinity Pulses off the Central Oregon Coast

**DOI:** 10.1038/srep17145

**Published:** 2015-11-26

**Authors:** Piero L. F. Mazzini, Craig M. Risien, John A. Barth, Stephen D. Pierce, Anatoli Erofeev, Edward P. Dever, P. Michael Kosro, Murray D. Levine, R. Kipp Shearman, Michael F. Vardaro

**Affiliations:** 1 Department of Marine and Coastal Sciences Rutgers, The State University of New Jersey, 71 Dudley Road New Brunswick, NJ 08901; 2 College of Earth, Ocean, and Atmospheric Sciences Oregon State University, 104 CEOAS Administration Building, 101 SW 26th St, Corvallis, OR 97331, United States.

## Abstract

From mid-May to August 2011, extreme runoff in the Columbia River ranged from 14,000 to over 17,000 m^3^/s, more than two standard deviations above the mean for this period. The extreme runoff was the direct result of both melting of anomalously high snowpack and rainfall associated with the 2010–2011 La Niña. The effects of this increased freshwater discharge were observed off Newport, Oregon, 180 km south of the Columbia River mouth. Salinity values as low as 22, nine standard deviations below the climatological value for this period, were registered at the mid-shelf. Using a network of ocean observing sensors and platforms, it was possible to capture the onshore advection of the Columbia River plume from the mid-shelf, 20 km offshore, to the coast and eventually into Yaquina Bay (Newport) during a sustained wind reversal event. Increased freshwater delivery can influence coastal ocean ecosystems and delivery of offshore, river-influenced water may influence estuarine biogeochemistry.

Annually, after the spring transition^1^, winds off the Oregon and Washington coast are predominantly southward (upwelling-favorable) and the Columbia River Plume (CRP) is advected to the south by the coastal upwelling jet, and offshore by the cumulative Ekman transport, detaching the plume from the coast. The shelf currents and wind forcing overwhelm the natural tendency of the plume to turn to the right due to the Coriolis effect, and as a result, relatively fresh water is observed off the Oregon coast^2^. Departures from this average state, however, can be quite dramatic, with a bi-directional CRP also often influencing the Washington shelf during summer^3^,^4^.

The location of plume water as well as its freshwater content are important to the Oregon-Washington shelf ecosystem as it affects sediment deposition, nutrient concentrations as well as circulation and stratification^3^. Recent work has shown the importance of the CRP to shelf ecosystems and biogeochemistry including increased primary productivity, enhanced macrozooplankton concentration, delivery of biologically important micronutrients, and offshelf chlorophyll export^5^,^6^,^7^,^8^.

Because rivers and estuaries differ in their carbon chemistry dynamics from the open shelf, there is increased interest in how these regions interact with respect to ocean acidification. River-influenced water on the open continental shelf can enter coastal estuaries and influence both the physics, including stratification and circulation, and biogeochemistry found there. Since the Columbia River is the largest freshwater source on the U.S. west coast, it is important to estimate the reach of its alongshore influence on both continental shelf ecosystems and coastal estuaries.

While a number of observations^9^,^10^,^11^,^12^,^13^ reveal frequent intrusions of the CRP into estuaries north of the Columbia River, along the Washington coast, to our knowledge observations of CRP waters entering estuaries south of the Columbia River have not been previously reported, despite the fact that the CRP may extend over 500 km south of the Columbia River mouth^2^.

From mid-May through August 2011, extreme Columbia River discharge reached more than two standard deviations above the mean for most of this period, having a peak discharge of nearly 17,000 m^3^/s in early June (Fig. 1c). This anomalous discharge as well as the atmospheric circulation and climate anomalies observed over the Pacific Northwest region during spring of 2011 can be explained by an extreme La Niña event^14^,^15^. The months of February and March reached the highest monthly Southern Oscillation Index (SOI) since 1866, while the month of April had the highest value since 1903 and the 2nd-highest on record. The impacts of this La Niña in the Pacific Northwest, with approximately a 3-month lag, were characterized by a longer storm season, increased spring snowfall in the Cascade Range, and negative temperature anomalies^14^. Just as extreme weather events influence terrestrial ecosystems and have potential economic impacts^16^, it is important to understand the impact of such events on marine ecosystems.

Effects of the anomalous discharge from the Columbia River on the coastal ocean thermohaline structure were observed offshore of Newport, Oregon, approximately 180 km south of the Columbia River mouth. Using a suite of sensors and platforms, including three moorings, land-based HF-radar, satellite remote sensing and underwater gliders, we describe the onshore advection of the CRP off Newport, OR, from the mid-shelf, nearly 20km offshore, to the coast and eventually into the Yaquina Bay (Newport) during a sustained wind reversal event.

In the next section we present our results, which are structured as follows: first the broader scale weather patterns are characterized as well as their effect on the CR streamflow during the spring/summer of 2011; then the spring transition and its effects on thermohaline structure of the coastal ocean are presented; lastly, a case study is presented, in which both satellite remote sensing and *in situ* moored observations are used to demonstrate the onshore propagation of the CRP onto the inner-shelf and into the Yaquina bay estuary and the consequent impact in the estuarine biogeochemistry. The results section is followed by the discussion section in which we place our findings in context and summarize and discuss our conclusions. Finally in the methods section we describe the data and methods used in this study.

## Results

### Extreme Columbia River Discharge

**Figure 1:**
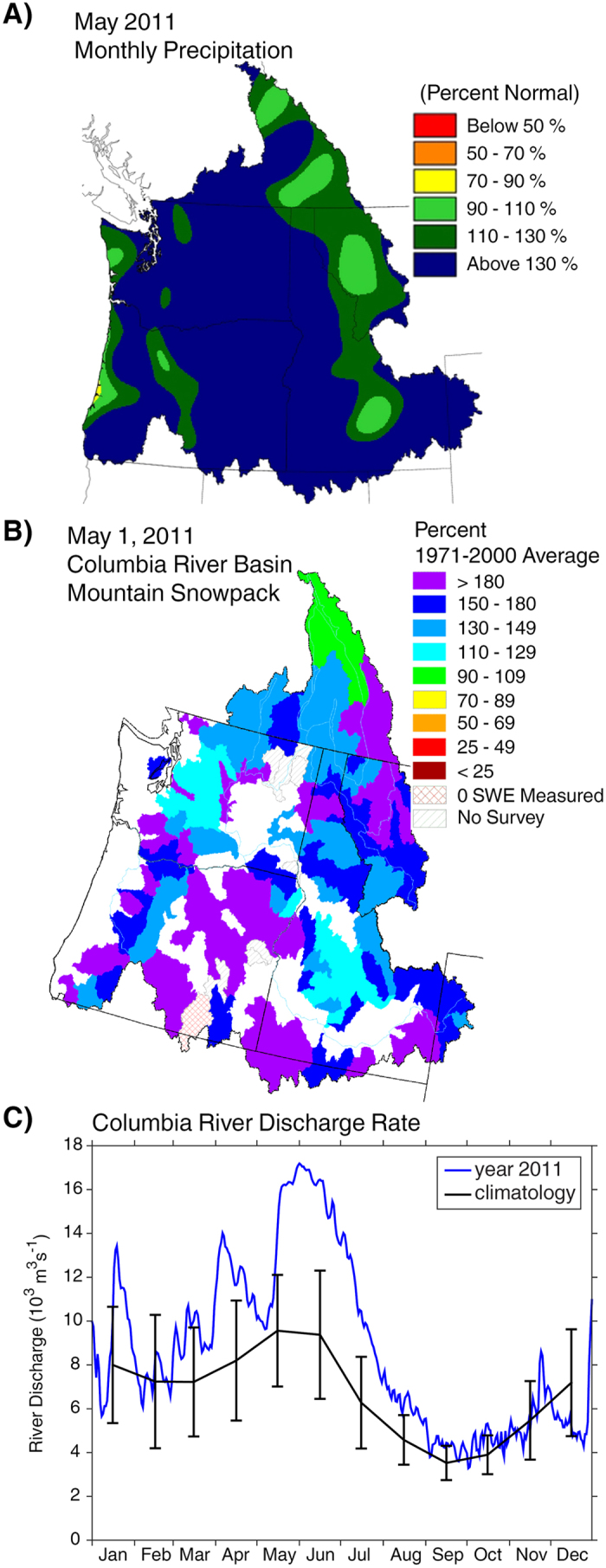
(**a**) Average precipitation across the Pacific Northwest for May 2011. Map obtained from the National Oceanic and Atmospheric Administration (http://www.nwrfc.noaa.gov/water_supply/sea_wy_summary.cgi?wy=2011). (**b**) Mountain snowpack across the Columbia River basin on 1 May 2011. Map obtained from the United States Department of Agriculture (http://www.wcc.nrcs.usda.gov/ftpref/support/snow/snowpack_maps/columbia_river/wy2011/cusnow1105.gif). (**c**) Columbia River discharge climatology (black), with standard deviations, based on the 20-year period 1993–2012. Daily mean Columbia River discharge rates for 2011 (blue).

Abnormally high precipitation rates and snow accumulation were observed in the Pacific Northwest during spring 2011. The May average precipitation map (Fig. 1a) shows values that are 130% of climatological mean, for most of the Columbia River drainage area. Snowpack (Fig. 1b) for most of the region had values ranging from 150–180% of the mean, and several places over 180% of the mean. As a result of snow melt from extremely high levels of snowpack and high precipitation rates during spring 2011, anomalously high Columbia River discharge rates were observed during the spring and summer 2011 (Fig. 1c). The first of two significant discharge peaks occurred from the end of March until the beginning of May, varying from 9,000 to 14,000 m^3^/s. The second, more significant peak, persisted from mid-May through August. During this period, discharge rates peaked at more than 17,000 m^3^/s, or 82% above the climatological monthly average. As a direct response to this anomalous discharge, surface salinity values as low as 22 were observed 18 km west of Newport, OR at NH-10 on 25–26 May (not shown), nine standard deviations below the climatological value for this period. These were also the lowest recorded values since sustained NH-10 mooring observations began in 1999.

**Figure 2:**
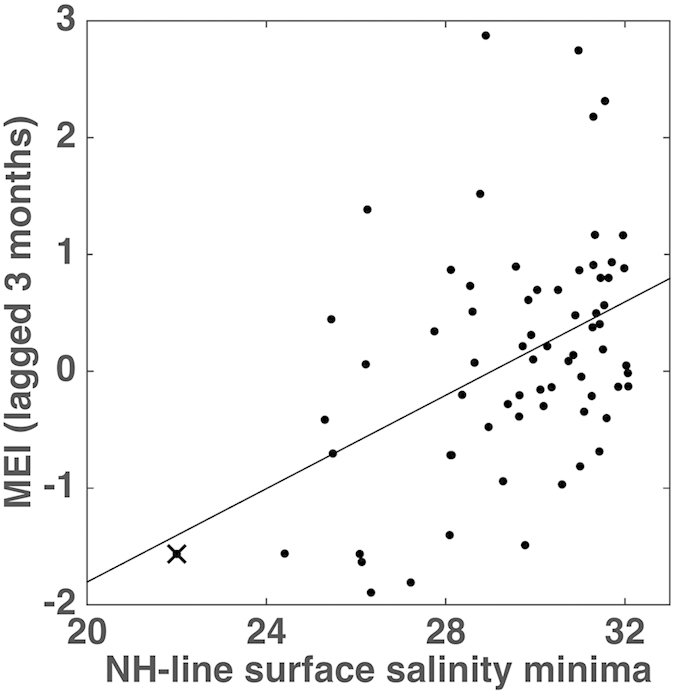
Monthly salinity minima lagged by 3 months, between 1959–2012 along the NH-line, plotted versus the Multivariate El Niño/Southern Oscillation Index (MEI)^19^,^20^. A regression line is plotted, with slope of 0.2 (+/−0.1). The lowest salinity measured at the NH-line, during spring 2011, is marked with an “x”.

La Niña events have been shown to influence streamflow in several regions of the USA, including the Pacific Northwest^17^,^18^. Ship observations from 1959–2007, complemented by glider data from 2006–2012, along the Newport Hydrographic (NH) Line, were analyzed to verify the impact of increased river discharge from the Columbia River due to La Niña events on salinity during the upwelling season. The Multivariate El Niño/Southern Oscillation Index (MEI)^19^,^20^, has a significant correlation with monthly salinity minima (r = 0.44, 95% confidence level of 0.39), and a regression is shown in Fig. 2, with an observed slope of 0.2 (+/−0.1). This analysis was conducted between the months of May and September. Only months which had a minimum of nine observations and reached at least 35 miles offshore were used.

### Thermohaline Structure and Freshwater Content

**Figure 3:**
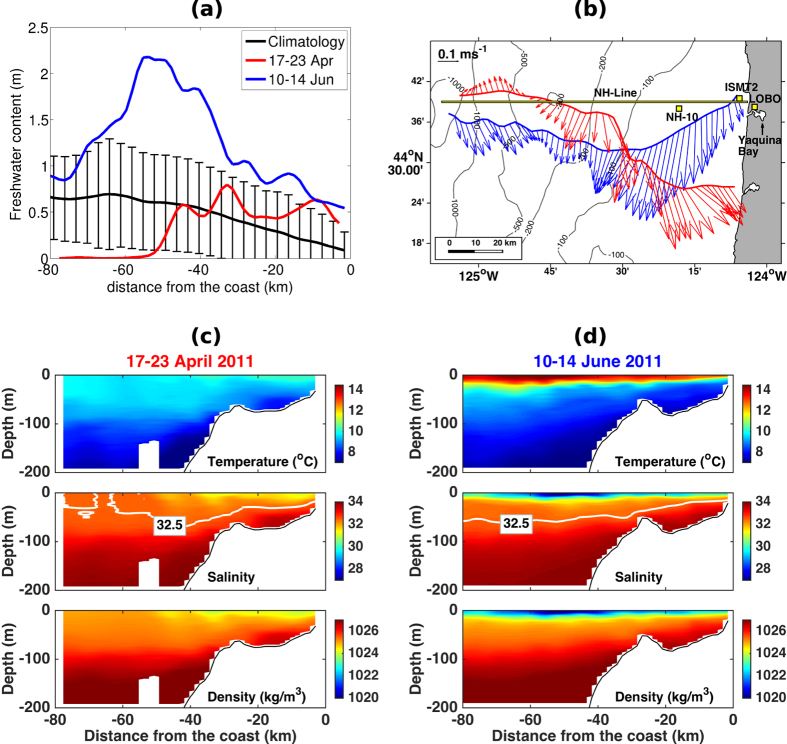
(**a**) Freshwater content in meters, as a function of cross-shelf distance, at the Newport Hydrographic line. In black, mean and standard deviations for upwelling seasons defined according to Pierce *et al*.^21^ calculated from underwater glider data from 2006–2012. In red, freshwater from a single glider transect, measured from 17–23 April 2011, and in blue, measured from 10–14 June2011. (**b**) Depth averaged velocities from glider transects off the NH-Line, and the location of the moorings: NH-10, ISMT2 and LOBO. Cross-shelf transects of temperature, salinity and density from glider transects, measured from 17–23 April 2011 (**c**) and 10–14 June 2011 (**d**).

In 2011, the upwelling season along the Oregon coast occurred from 16 April to 11 September, following the upwelling season definition in Pierce *et al*.^21^. In Fig. 3a, freshwater content as a function of cross-shelf distance as well as salinity, temperature, density and depth-averaged currents are shown for two glider transects: one at the beginning of the upwelling season, surveyed from 17–23 April 2011 (Fig. 3c), right before the high Columbia River discharge peak, and the second one later in the upwelling season, from 10–14 June 2011 (Fig. 3d), close to the discharge peak. For comparison, the mean and standard deviations of freshwater content estimated for the upwelling season using 272 salinity transects from 2006–2012 are also shown in Fig. 3a.

Freshwater content of the water column at the beginning of the upwelling season is confined between the coast and 50 km offshore, varying from 0.5–0.8 m, within one standard deviation from the mean for this time of the year. Later in the season, the second glider transect shows the presence of freshwater along its entire track, from the coast to 80 km offshore. Most of the freshwater content is above one standard deviation from the mean, with a maximum peak of 2.2 m, more than two standard deviations from the mean, centered around 55 km offshore.

Both glider transects show equatorward flow on the continental shelf (Fig. 3b), with maximum depth averaged velocities reaching over 0.4 m/s near the 80 m isobath, and then decreasing to about 0.1 m/s towards the coast and the shelf break. These are typical characteristics of the coastal upwelling jet observed off the Oregon coast^22^. It is also possible to see the isopycnals tilted upward towards the coast as a direct result of upwelling secondary circulation, bringing deeper denser water masses up to shallower regions. Figure 3c,d show that below 15–20 m depth, salinity and density fields from the two glider transects are remarkably similar, with vertical variations in salinity and density ranging from 32–34, and 1025–1027 kg/m^3^, respectively. Temperature profiles vary from 6.5–10 ^o^C, with slightly warmer temperatures (by 0.5–1 ^o^C) from 20–80 m at the beginning of the season. A pycnocline is observed between 1025.5–1026 kg/m^3^ situated from 70–90 m depth at the offshore extent of the transects. Inshore the pycnocline shallows to 20–50 m depth. These observations are consistent with historical NH-Line data^23^,^24^,^25^.

Major differences between the glider transects occur, however, in the upper 15–20 m when from 17–23 April 2011 (Fig. 3c) the water column had temperatures of approximately 9.5–10 °C along the entire transect, salinity of nearly 32.5 and density of 1025 kg/m^3^ offshore of the shelf break, where the water column was vertically homogeneous above 80–100 m. In contrast, over the shelf, the salinities were as low as 31 and densities were 1024 kg/m^3^. From 10–14 June 2011 (Fig. 3d), the upper 15–20 m had values ranging between 11–14.7 ^o^C in temperature, 27–32 in salinity, and 1020–1024 kg/m^3^ in density, with a very sharp transition to deeper waters, presenting another pycnocline besides the one observed in from 70–90 m.

### Frontal cross-shelf propagation

**Figure 4:**
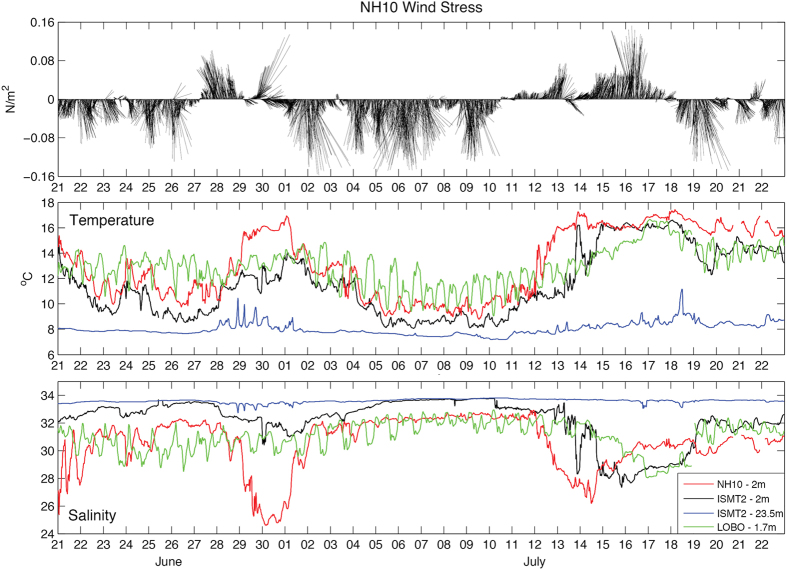
Time series of: wind stress from NOAA buoy station 46094 at NH-10, located approximately 2 km south of the NH-line (top); temperature (middle) and salinity (bottom) measured near surface (~2m) at NH-10, ISMT2 and LOBO, and at depth (23.5 m) at ISMT2.

Time series measurements of: windstress (estimated from wind velocities) from NOAA buoy station 46094 at NH-10, near surface (2 m depth) salinity and temperature at NH-10, ISMT2 and LOBO, and near-bottom (23.5 m depth) salinity and temperature at ISMT2 for 21 June to 23 July 2011 are presented in Fig. 4. NH-10 is located at the 81 m isobath (18 km offshore of Newport), ISMT2 is on the inner-shelf at the 25 m isobath, and LOBO is located inside Yaquina Bay estuary approximately 3.5 km from the estuary mouth (see Fig. 3b for mooring locations).

Figure 4 (top) shows that while northerly, upwelling-favorable, winds were blowing majority of the time between 21 June and 23 July 2011, as is commonly observed during this time of year [e.g.^26^], two significant wind reversal events did occur during this period. The first reversal started on 27 June and lasted 2.5 days and the second event started on 10 July and lasted 7.5 days.

High-frequency variability (periods less than a day) inside Yaquina Bay at LOBO, show oscillations in temperature (Fig. 4 middle) that can vary from to 2–4 ^o^C and salinity (Fig. 4 bottom) from 2–3, with a clear semi-diurnal tidal signal. Over the continental shelf, high frequency variability and the influence of tides are much smaller, with temperatures varying typically less than 0.5 °C and salinities less than 0.1.

Low-frequency variability (periods longer than a day) has a smaller effect inside Yaquina Bay, however it seems to dominate the signal over the continental shelf surface waters. Both surface temperature and salinity respond to the wind reversals, when through Ekman transport, warmer and fresher waters from offshore are advected towards the coast. Near bottom salinities and temperatures at ISMT2 have approximately constant values, showing small variations only during wind reversal events, typically less than 1 and 2 ^o^C, respectively, demonstrating that the wind effect on temperature and salinity is mainly confined to surface waters.

Approximately a half day after the first wind reversal event (27–30 June), surface temperatures at NH-10 and ISMT2 start to increase from 10.5 ^o^C and 9.5 ^o^C, respectively, and reach a maximum three days later, of 17 ^o^C and 13.5 ^o^C, respectively. Simultaneously, responses in salinities occur at both places, decreasing from 32 to 24.5 at NH-10, and from 33 to 30.5 at ISMT2. After winds veer to the south, temperature and salinity at both stations go back to their previous state before the reversal, at a comparable rate. The effects of the wind reversal in the Yaquina Bay are much smaller, and mostly masked by the tides. The larger decrease in salinity, of nearly 7.5 at NH-10 compared to only 2.5 at ISMT2, suggests that the CRP core is located offshore of NH-10, during upwelling favorable winds.

**Figure 5:**
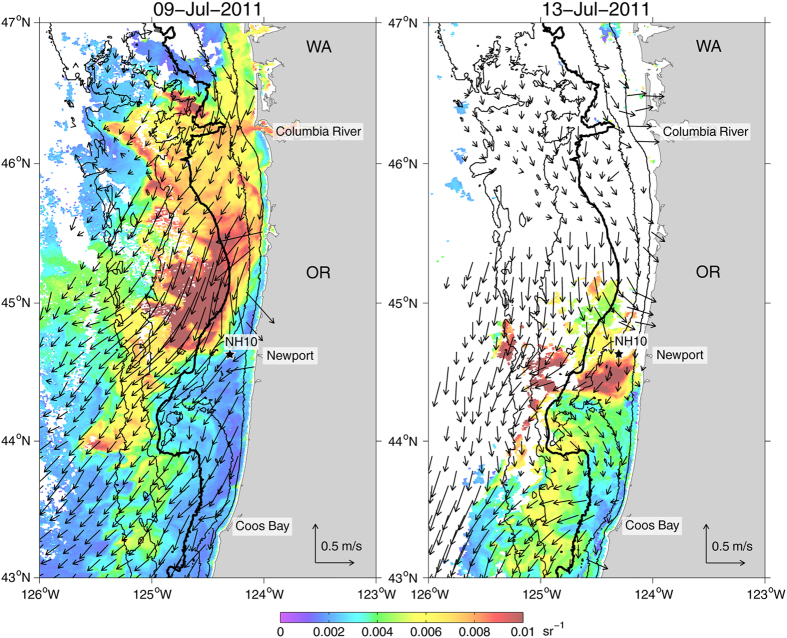
Daily averaged MODIS/Aqua 555 nm Rrs measurements, an effective tracer of particulate matter in the water column^47^, for 9 July 2011 (left) and 13 July 2011 (right) in color. Vectors show daily averaged detided surface current velocities derived from HF Radar observations. The isobaths of 50, 100, 200, 1000 and 2000-m are plotted in black, with the shelf break, indicated by the 200-m isobath, in thick black.

On 9 July, MODIS/Aqua 555 nm remote sensing reflectance (Rrs) measurements (Fig. 5, left) show the CRP being advected southward by upwelling favorable winds and surface currents with plume water located to the west of NH-10 and possibly as far south as 43 °N, 360 km south of the Columbia River mouth. Four days later, on 13 July (Fig. 5, right), the CRP has moved inshore of NH-10. This onshore movement coincides with the second 7.5 day wind reversal that occurred from 10–18 July. Approximately 1–1.5 days after the wind reversed, salinity at NH-10 started to decrease from 33 reaching a minimum of 26.3 about 2.5 days later. Decreases in salinities were also observed at ISMT2 from 33 to 27.5 and at LOBO from 30–32 to 28.2, 1.3 and 2.2 days after NH-10 local minimum, respectively (Fig. 4 bottom), showing the intrusion of the CRP into Yaquina Bay. Similarly, increases in temperature occurred from 10 to 17.3 ^o^C at NH-10, from 8 to 16 ^o^C at ISMT2, and from 12 to 16.5 ^o^C at LOBO (Fig. 4 middle).

While the observed minimum salinity values in the Yaquina Bay estuary during the intrusion of the CRP (Fig. 4 bottom) may not be much lower than the typical variability observed during other periods (e.g., 0.5 PSU difference on 26 June 2011, Fig. 4), the estuary is exposed to low salinity for periods longer than one tidal cycle when the CRP is present.

### Effect of the CRP intrusion on Yaquina Bay biogeochemistry

Besides impacting the salinity field, the intrusion of the CRP into Yaquina Bay also affects the estuary’s biogeochemistry. Available nutrient data collected at the Oregon State University dock in Yaquina Bay during the CRP intrusion and afterward, are shown in Table 1. The lowest concentrations of both phosphate (0.61 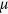
M) and nitrate +nitrite (0.78 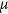
M) occur on 16 July 2011, nearly 2 days after the beginning of the CRP intrusion (Fig. 4 bottom), while no apparent effect is observed on ammonium concentrations. On 21 July 2011, nearly 3 days after wind reversed to upwelling-favorable and following a retreat of the CRP from Yaquina Bay (marked by the increase in salinity, Fig. 4 bottom), concentrations of phosphate increased by nearly 50% while nitrate+nitrate increased by over 200%. These nutrient levels continue to increase as time progresses, with phosphate reaching values as high as 2.36 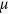
M and nitrate+nitrite as high as 24 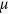
M on 27 July 2011, nearly 8 days after the complete receding of the CRP.

## Discussion

The input of freshwater into the coastal ocean can significantly influence local dynamics, mainly by increasing near-surface stratification and creating horizontal density gradients that can generate currents. In addition to that, river plumes transport larvae, nutrients, carbon, sediments and pollutants from the estuaries onto the continental shelves, playing an important role in marine biogeochemistry^7^. Therefore, it is important to monitor and understand what mechanisms influence the spatial and temporal variability of freshwater in the ocean.

After the spring transition^1^, waters from the CRP are mostly advected to the south, which creates strong stratification in the ocean upper layer (15–20 m deep) off the Oregon coast. This sharp pycnocline created from the presence of the CRP waters inhibits vertical motions and therefore vertical mixing in the ocean, isolating the upper layer from the rest of the water column. Solar radiation is an important heat source at this time of the year, and since waters above 15–20 m are isolated from the rest of the water column, heating is confined mainly to the upper ocean, which further decreases its density and increases stratification [e.g.^27^]. The anomalous increase of discharge from the Columbia River can be traced by the freshwater content along the NH-line, which plays a key role in controlling the shelf stratification and further potentially acting on thermodynamics processes over the continental shelf. While other river plumes have been shown to influence thermodynamics processes in different regions of the ocean [e.g.^28^,^29^], the effect of the CRP off Oregon and Washington still needs to be investigated.

Based on the distance from the Columbia River mouth to the NH-Line (180 km), and the depth averaged velocities observed from the gliders (~0.4 m/s), an estimated time response for the Columbia River discharge based on an advective time scale would be 5.2 days to impact the NH-Line. This is perhaps an underestimation, since most of the Columbia River waters are within the top ~20 m of the water column, and the upwelling jet is surface intensified^30^. Surface velocities estimated from HF-radar reveal that the coastal jet core can reach over 1 m/s^22^, which would give a time response as fast as 2 days.

Despite the fact that CRP may extend over 500 km south of the Columbia River mouth^2^, our observations show for the first time the CRP cross-shelf propagation from mid-shelf all the way to the coast, entering the Yaquina Bay estuary. Evidence from Kirincich and Barth^31^, and Adams *et al*.^32^, suggests that the CRP may propagate inshore and reach the inner-shelf about 1–3 times per month during the upwelling season. Based on the time series shown in Fig. 4 (between 14 and 17 July 2011), the surface front propagated nearly 20 km in about 2.2 day, which gives an average speed of 0.1 m/s. If we assume that the alongshore momentum is in Ekman balance to first order^33^, that the Ekman layer is confined to the plume thickness, and assuming a linear profile for the velocity within the Ekman layer^34^, the velocity at the surface can be calculated as 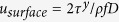, where 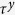 is the wind stress parallel to the coast, 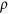 is the water density, 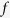 is the Coriolis parameter and 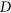 is the plume thickness. From observations, if we use 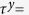
0.08 N/m^2^, 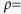
1024 kg/m^3^, 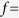
10^−4^ s^−1^ and 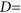
15–20 m, we would obtain velocities between 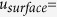
0.08–0.1 m/s, which are consistent with the time series observations. Other more complex models that predict the response of river plumes to wind-forcing [e.g.^35^], could not be tested here, as they require more detailed information about, for example, the plume cross-sectional area. Nevertheless, with this simple 2D model, the cross-shelf propagation of the CRP off central Oregon can be explained in terms of Ekman dynamics.

An important result presented here is the considerable alongshore reach of CRP water over the continental shelf and its subsequent movement into coastal estuaries such as the Yaquina Bay. Relative to winter, summer discharge rates of Oregon’s coastal rivers, not including the Columbia River, are significantly lower. For example, the average Yaquina River discharge rates for January and July are 17.48 (+/−7.48) and 0.83 (+/−0.38) m^3^/s, respectively. This reduction in river discharge and the fast tidal flushing of the Yaquina Bay, a small volume estuary with large tidal prism, results in its hydrographic and nutrient variability being dominated by coastal ocean processes during the summer season^36^,^37^.

North of the Columbia River mouth, along the Washington coast, Hickey *et al*.^38^ have shown that residual estuarine circulation is impacted by downwelling (upwelling) events, even in the absence of the CRP. These events result in a decrease (increase) in density near the estuary mouth, modifying along-estuary baroclinic pressure gradients. The presence of the CRP during the downwelling events acts to further decrease the density near the estuary mouth, enhancing the impact on the residual estuarine circulation. While it has been demonstrated here that CRP waters also impact Oregon’s estuaries during downwelling-favorable winds, its influence on the estuarine dynamics is expected to be smaller than that observed off Washington, since the CRP has first gone through upwelling (more mixing), and therefore it is an older and saltier plume, with a smaller density contrast^10^.

The biogeochemical data presented here are consistent with the results presented in Sigleo *et al*.^36^ and Brown & Ozretich^37^, which demonstrate that the ocean is the main source of nutrients to Yaquina Bay during the dry season (May to October), and that increases (decreases) of nutrient concentrations are observed during sustained periods of upwelling-favorable (downwelling-favorable) winds. While far-field CRP waters have low concentrations of macronutrients^39^, Lohan & Bruland^5^ and Brown & Bruland^6^ show that this far-field CRP water contains elevated levels of micronutrients such as dissolved and particulate aluminium, iron and silicic acid. While no direct micronutrient measurements are presented here, these have been shown to influence the species composition, growth rates, and final yields of Yaquina Bay phytoplankton^40^. Another important characteristic of Columbia River waters is that the pH is generally lower than seawater^41^, potentially contributing to acidification of Yaquina Bay. Thus the movement of Columbia River water into coastal estuaries not only provides a pathway for the movement of organisms between estuaries but it also suggests a mechanism by which remote estuarine waters with their own particular biogeochemical composition can enter and influence the biogeochemistry of local estuaries many of which, including Yaquina Bay, support shellfish farming that is estimated to contribute over $270 million in regional economic activity^42^.

Finally, the case study presented here demonstrates that ocean observatories, such as NOAA IOOS^43^ and the recently deployed Ocean Observatories Initiative^44^, have great value for marine science and for coastal management, since they allow us to monitor and understand the coastal ocean by resolving processes both spatially and temporally. It is important to combine both surface and subsurface measurements, both *in situ* and remotely sensed, from a variety of sensors measuring physical and biogeochemical ocean properties. When compared with historical data sets, near real-time ocean observatories can be used to detect anomalous events. In the future, ocean observatories will provide continuous information to help understand climate change and its impacts on the marine and coastal environments.

## Methods

The glider data were provided by the glider research group from Oregon State University (http://gliderfs2.coas.oregonstate.edu/gliderweb). Surveys were conducted at the Newport Hydrographic Line (NH-line, 44°39′N), between 2–80 km from the coast using Slocum gliders from Teledyne Webb Research^45^. Data were collected from temperature, conductivity and pressure sensors (Sea-Bird Electronics, Inc. SBE-41CP) onboard the gliders, as well as depth-averaged horizontal velocities calculated from dead reckoning. A more detailed description of this data set and processing can be found in Mazzini *et al*.^46^.

NH-line shipboard surface salinity data from 1959–2007 were obtained from the World Ocean Database 2013 (WOD13), with exclusion using all National Oceanographic Data Center (NODC) quality control flags.

The Inshore Mooring Test 2 (ISMT2) data was provided by the Ocean Observatories Initiative (OOI). The mooring was located at 44^o^39′29.9″N, 124°05'44.1″W, at the 25 m isobath, between 19 March and 6 August 2011. Data come from two sensors of temperature, conductivity and pressure sensors (Sea-Bird Electronics, Inc. SBE-16plus) installed near surface around 2 m deep, and near bottom, at approximately 23.5 m deep.

Hourly time series data from Land/Ocean Biogeochemical Observatory (LOBO), located inside the Yaquina Bay estuary, at 44°37′45.82″N 124^o^2′29.25″W, are available at http://yaquina.loboviz.com. The data used in this analysis come from temperature, conductivity and pressure sensors (Sea-Bird Electronics, Inc. and WET Labs, Inc., Water Quality Monitor - WQM).

Surface currents derived from land-based high-frequency radars were made available from the Ocean Currents Mapping Lab from Oregon State University, http://currents.coas.oregonstate.edu. The data are collected hourly by an array of 11 shore-based HF-radars from Loomis Lake, WA (46°26′16.8″N) to Crescent City, CA (41°47′41.78″N), then the data are detided and daily averaged, with a spatial resolution of 6 km.

NH-10 data was provided by the Northwest Association of Networked Ocean Observing Systems (NANOOS). The mooring was located at 44°38′N, 124°18.2′W, at the 81 m isobath. Data used in the work come from temperature, conductivity and pressure sensors (Sea-Bird Electronics, Inc. SBE-37) installed near the surface around 2 m deep, and from a meteorological station at the surface buoy, which is also available as NOAA National Data Buoy Center buoy 46094.

MODIS satellite data were from level-2, with 1 km resolution, obtained from OceanColorWEB (http://oceancolor.gsfc.nasa.gov/), maintained by NASA.

The Multivariate El Niño/Southern Oscillation Index (MEI) is made available by NOAA at http://www.esrl.noaa.gov/psd/enso/mei/#ref_wt1.

The freshwater content F_s_ in meters, was calculated following Mazzini *et al*. [2014]: 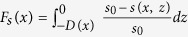 , where 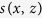 is the measured salinity, 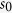 is the reference salinity, and 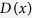 is the depth at which it occurs. The value chosen for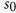 was 32.5 following Barnes *et al*.^2^.

## Additional Information

**How to cite this article**: Mazzini, P. L. F. *et al*. Anomalous Near-Surface Low-Salinity Pulses off the Central Oregon Coast. *Sci. Rep*. **5**, 17145; doi: 10.1038/srep17145 (2015).

## Figures and Tables

**Table 1:** Nutrient concentrations in 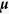 M, obtained from measurements at the Oregon State University dock at Yaquina Bay, Newport, Oregon.

Date	Phosphate	Nitrate+Nitrite	Ammonium
7/16/11 18:15	0.62	0.77	2.10
7/16/11 18:15	0.59	0.79	1.98
7/21/11 11:15	0.89	1.82	2.20
7/21/11 11:15	0.87	1.77	2.14
7/27/11 9:00	2.00	24.00	1.67
7/27/11 9:00	2.36	24.00	1.62
8/11/11 8:15	1.90	17.60	1.65
8/11/11 8:15	1.86	17.40	1.85
